# Deep Grey Matter Volume is Reduced in Amateur Boxers as Compared to Healthy Age-matched Controls

**DOI:** 10.1007/s00062-022-01233-3

**Published:** 2022-12-16

**Authors:** Mousa Zidan, Jessica Jesser, Christian Herweh, Joachim Jost, Sabine Heiland, Uta Meyding-Lamadé, Martin Bendszus, Stefan Haehnel

**Affiliations:** 1 Department of Neuroradiology, Heidelberg University Hospital, Im Neuenheimer Feld 400, 69120 Heidelberg, Germany; 2 National Training Center for Boxing, Heidelberg, Germany; 3 Division of Experimental Radiology, Department of Neuroradiology, Heidelberg University Hospital, Heidelberg, Germany; 4 Department of Neurology, Krankenhaus Nordwest, Frankfurt, Germany

**Keywords:** Mild traumatic brain injury, Dementia, Parkinson’s disease, Diffuse axonal injury, Concussion

## Abstract

**Purpose:**

Mild traumatic brain injuries (mTBI) sustained during contact sports like amateur boxing are found to have long-term sequelae, being linked to an increased risk of developing neurological conditions like Parkinson’s disease. The aim of this study was to assess differences in volume of anatomical brain structures between amateur boxers and control subjects with a special interest in the affection of deep grey matter structures.

**Methods:**

A total of 19 amateur boxers and 19 healthy controls (HC), matched for age and intelligence quotient (IQ), underwent 3T magnetic resonance imaging (MRI) as well as neuropsychological testing. Body mass index (BMI) was evaluated for every subject and data about years of boxing training and number of fights were collected for each boxer. The acquired 3D high resolution T1 weighted MR images were analyzed to measure the volumes of cortical grey matter (GM), white matter (WM), cerebrospinal fluid (CSF) and deep grey matter structures. Multivariate analysis was applied to reveal differences between groups referencing deep grey matter structures to normalized brain volume (NBV) to adjust for differences in head size and brain volume as well as adding BMI as cofactor.

**Results:**

Total intracranial volume (TIV), comprising GM, WM and CSF, was lower in boxers compared to controls (by 7.1%, *P* = 0.009). Accordingly, GM (by 5.5%, *P* = 0.038) and WM (by 8.4%, *P* = 0.009) were reduced in boxers. Deep grey matter showed statistically lower volumes of the thalamus (by 8.1%, *P* = 0.006), caudate nucleus (by 11.1%, *P* = 0.004), putamen (by 8.1%, *P* = 0.011), globus pallidus (by 9.6%, *P* = 0.017) and nucleus accumbens (by 13.9%, *P* = 0.007) but not the amygdala (by 5.5%, *P* = 0.221), in boxers compared to HC.

**Conclusion:**

Several deep grey matter structures were reduced in volume in the amateur boxer group. Furthermore, longitudinal studies are needed to determine the damage pattern affecting deep grey matter structures and its neuropsychological relevance.

## Introduction

Mild traumatic brain injury (mTBI) is very common among athletes performing contact sports, like amateur boxing, but also military personnel and older people can be affected, with an estimated number of 42 million cases worldwide each year [1]. The evidence concerning supposed mTBI in amateur boxers has been inconsistent and previous studies have delivered contradictory findings; some reported diminishing of cognitive functions [2, 3], some proved through the analysis of biomarkers in cerebrospinal fluid (CSF) strong evidence of traumatic axonal injury even without measurable cognitive impairment [4], whereas others were skeptical about the presence of credible evidence connecting amateur boxers to chronic brain injury [5–7].

During the acute phase after mTBI, following the definition of Orman et al. [8], loss of consciousness should not be longer than 30 min, post-traumatic amnesia should not last longer than 1 day, and the Glasgow Coma Scale (GCS) cannot be below 13. In terms of imaging criteria, it is commonly acknowledged that to fulfill the definition of mTBI only minor or no structural changes, like contusions or microhemorrhages, should be detectable on conventional CT or MRI (magnetic resonance imaging) after the traumatic incident [9]. Instead of obvious structural changes, more subtle changes occur in mTBI, such as diffuse axonal injury, which can be detected by diffusion tensor imaging (DTI) after mTBI [10, 11] also in amateur boxers [12], and cortical thinning, assessed by high-resolution (HR) structural MRI [13, 14]. Also, deep grey matter structures were shown to be affected by volume loss after repeated head trauma in professional fighters. Lower brain volumes in the thalamus and caudate nucleus and less pronounced in the putamen were associated with years of fighting and fight exposure [15]. There is only a limited number of studies exploring the affection of deep grey matter after traumatic brain injuries let alone mTBI. Nevertheless, in recent years mTBI has gained the spotlight for its role in initiating long-term neurodegeneration cascades which result in neurodegenerative diseases also affecting the basal ganglia like Parkinson’s disease [16].

The main objective of this study is to evaluate the effect of amateur boxing on deep grey matter volumes compared to age-matched healthy controls.

## Subjects and Methods

### Study Design and Subjects

This prospective study was approved by the institutional ethics board and written informed consent was obtained from all participants prior to the examinations.

Included in this study were 19 male amateur boxers recruited from the local Olympic base as well as 19 male healthy control subjects (HC) with no history of any sort of contact sport or boxing. Boxers and HC were chosen from a previously published cohort [17] and matched for age and intelligence quotient (IQ). Participant recruitment, imaging and neuropsychological data collection took place in 2005 [17]. Amateur boxers have full contact in training whilst wearing protective headgear. Exclusion criteria for boxers and HC were anamnestic or imaging evidence for previous moderate or severe traumatic brain injury as defined by Orman et al. [8], participation in any other forms of contact sports (e.g., American football, soccer), any neurological or psychiatric diseases, metabolic diseases, arterial hypertension, substance abuse or any accidental findings which may have interfered with or influenced brain volumetry (e.g., arachnoid cyst and intercranial masses). Subjects with MRI contraindications (e.g., body implants not considered to be 3T MRI-safe) were also excluded.

The revised Hamburg Wechsler Intelligence Examination (HAWIE-R) [18] was performed and referred to as the intelligence quotient (IQ). In addition, planning, alertness and memory performances were assessed using the subtest “alertness” from the “test of attentional performance” (TAPAL) [19, 20], parts A and B of the trail making test (TMT‑A and TMT‑B, respectively) and the verbal learning and memory test (VLMT) [21].

Weight and BMI (body mass index) were assessed for all study subjects. Total number of fights as well as time extent of boxing (since beginning of training) were assessed for boxers.

### MR Data Acquisition

All subjects underwent the same MRI protocol on a 3T MR scanner (TRIO; Siemens, Erlangen, Germany) with an 8‑channel head coil. The MRI protocol consisted of transverse dual spin-echo MR imaging sequence (TR/TE, 5850/10.90 ms; section thickness, 6 mm; flip angle, 149°; acquisition matrix, 256 × 256; FOV, 200 mm), a 3D sagittal magnetization-prepared rapid acquisition of gradient echo (MPRAGE) sequence (TR/TE, 2250/3 ms; section thickness, 1 mm; flip angle, 9°; acquisition matrix, 256 × 256; FOV, 245 mm), a coronal T2*-weighted sequence (TR/TE, 599/20 ms; section thickness, 6 mm; flip angle, 20°; acquisition matrix, 256 × 256; FOV, 200 mm), and an axial time-of-flight (TOF) MR angiography sequence (TR/TE, 42/4.67 ms; section thickness, 0.8 mm; flip angle, 25°; acquisition matrix, 512 × 512; FOV, 200 mm).

### MR Image Volumetry

Each set of 3D MPRAGE images was converted from digital imaging and communication in medicine (DICOM) into Neuroimaging Informatics Technology Institute (NIFTI) format and uploaded onto the pipeline volBrain (https://volbrain.upv.es) [22]. It is based on the following preprocessing steps: denoising using the spatially adaptive non-local-means (SANLM) filter [23], coarse inhomogeneity correction [24], registration to Montreal Neurological Institute (MNI) space [25], fine inhomogeneity correction using statistical parametric mapping (SPM) [26], intensity normalization [27]. After that, further steps follow to aid the estimation of brain volumes at different scales: non-local intracranial cavity extraction (NICE) [28], tissue classification [29], non-local hemisphere segmentation (NABS) [30] and non-local subcortical structure segmentation [31].

For this investigation we measured total intracranial volume (TIV), defined as the sum of intracranial grey matter (GM), white matter (WM) and cerebrospinal fluid (CSF), normalized brain volume (NBV), defined as the sum of GM and WM divided by TIV, as well as deep grey matter volumes of the thalamus, globus pallidus, putamen, caudate nucleus, nucleus accumbens and amygdala, respectively. Volume values for both sides were added.

### Statistical Analysis

All statistical analyses were performed using SPSS (Version 24; SPSS Inc, Chicago, IL, USA).

Subject characteristics were summarized using descriptive statistics and are presented as mean standard deviation (STD). *P* values ≤ 0.05 were regarded as statistically significant.

Student’s t‑tests were used to assess differences between the boxer and HC group for TIV, GM, WM and NBV. Correlation analyses between BMI and TIV, GM and WM were conducted to assess interactions between those variables.

**Fig. 1:**
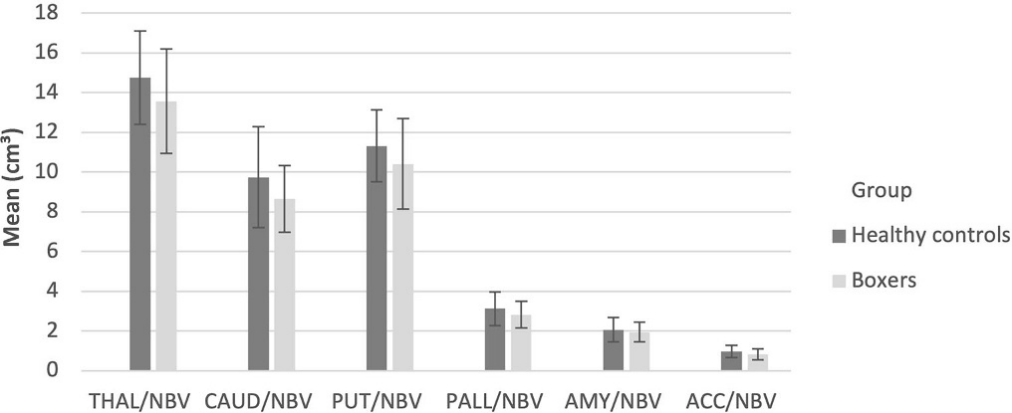
Deep grey matter structure volumes. Error bars indicate ± two standard deviations. *NBV* normalized brain volume, *THAL* thalamus, *CAUD* caudate nucleus, *PUT* putamen, *PALL* globus pallidus, *AMY* amygdala, *ACC* nucleus accumbens

For statistical analysis of the deep grey matter, these structures were set into reference to head size by dividing the volume of the structure by NBV, analogous to the methods used in Ward et al. [32] as presented in Fig. 1. Deep grey matter structures were compared between the boxer and HC groups by applying a multivariate analysis of variance with BMI. Our results were also corrected for effects of age in an analysis of covariance. Linear regression analysis examined the association between number of fights and duration of boxing in years on regional brain volumes of boxers.

## Results

### Boxer and Healthy Control Subject Characteristics

In the boxer group mean age was 29.1 ± 14.3 years (STD in years; median, 22 years; range, 17–65 years), mean BMI was 23.3 ± 3.4 (STD; median, 22.6; range, 18.8–31.3). For HC mean age was 29.4 ± 12.6 years (STD in years; median, 24 years; range, 16–26 years), mean BMI was 23.5 ± 2.9 (STD; median, 23.0; range, 18.8–29.6).

The mean number of fights in the boxer group was 89.2 ± 62.1 (STD; median, 76; range, 0–216). The mean time extent of boxing was 8.9 ± 4.8 years (STD in years; median, 10 years; range, 3–22 years).

Results of the neuropsychological testing are presented in Table 1. There were no significant differences in testing results between the boxer and HC group.

**Table 1:** Neuropsychological test results and between group comparisons

Neuropsychological test	Boxers (mean ± STD)	Healthy controls (mean [cm^3^] ± STD)	*P*-value (t-test)
*IQ [pts]*	117.6 ± 12.4	119.9 ± 8.5	0.51
*TMT‑A [s]*	26.9 ± 9.1	29.9 ± 12.4	0.64
*TMT‑B [s]*	57.9 ± 12.9	55.8 ± 16.0	0.66
*VLMT [pts]*	55.3 ± 9.9	60 ± 10	0.12
*VLMT_R1 [pts]*	12.2 ± 2.5	12.6 ± 2.4	0.64
*VLMT_R2 [pts]*	11.9 ± 3.1	12.5 ± 2.9	0.54
*TAPAL [s]*	204.2 ± 19.5	214.3 ± 31.8	0.45

Results of the student’s t‑test analysis for neuropsychological test results

*pts* points, *IQ* intelligent quotient, *TMT* trail making test, *VLMT* verbal learning and memory test, *TAPAL* test of attentional performance and alertness, *STD* standard deviation

### Structural MRI and MR-Volumetry Results

In our amateur boxer group, no subject showed signs of intracranial traumatic brain injuries, like microhemorrhages or parenchymal lesions. One boxer subject had a small (2 mm) aneurysm located at the anterior communicating artery.

Kolmogorov-Smirnov and Shapiro Wilk tests confirmed the normal distribution of our volumetric measurements.

The TIV, GM and WM volumes were significantly lower in the boxer group than in the HC group. Means and standard deviations as well as the mean difference in percent and the results of the statistical comparison by Student’s t‑test can be found in Table 2.

**Table 2:** Total intracranial, brain, grey and white matter volumes and between group comparisons

Structure for volumetry	Boxers (mean [cm^3^] ± STD)	Healthy controls (mean [cm^3^] ± STD)	difference in %	*P*-value (t-test)
*Total intracranial volume*	1440.0 ± 126.3	1550.0 ± 124.8	7.1	**0.009**
*Total brain volume*	1091.7 ± 107.4	1174.1 ± 89.0	7.0	**0.009**
*Total grey matter volume*	736.7 ± 64.1	779.7 ± 62.4	5.5	**0.038**
*Total white matter volume*	523.6 ± 67.7	572.2 ± 50.9	8.4	**0.009**

Results of Student’s t‑test analysis

*STD* standard deviation

Values of *p* < 0.05 are considered statistically significant and are marked in bold

The BMI significantly correlated with TIV (Pearson’s r = 0.42, *P* = 0.009), with NBV (r = 0.46, *P* = 0.003) as well as with total WM volume (r = 0.38, *P* = 0.019), but not with total GM volume (r = 0.06; *P* = 0.721).

A multivariate analysis of deep grey matter structure volumes with BMI as a covariate and post hoc testing revealed significantly lower volumes in the boxer group for the caudate nucleus, the putamen, the globus pallidus and the nucleus accumbens but not for the amygdala. In absolute values the difference in volume was most pronounced for the nucleus accumbens with 13.9% less volume in the boxer group compared to the HC group. Results of the analysis are summarized in Table 3. In addition, our results retained significance when corrected for the effect of age.

**Table 3:** Multivariate analysis of deep grey matter structure volumes

Structure for volumetry	Boxers (Mean [cm^3^] ± STD)	Healthy controls (Mean [cm^3^] ± STD)	difference in %	*P*-value of post hoc test
*Thalamus*	13.6 ± 1.3	14.8 ± 1.2	8.1	**0.006**
*Caudate nucleus*	8.7 ± 0.8	9.7 ± 1.3	11.1	**0.004**
*Putamen*	10.4 ± 1.1	11.3 ± 0.9	8.1	**0.011**
*Globus pallidus*	2.8 ± 0.3	3.1 ± 0.4	9.6	**0.017**
*Amygdala*	2.0 ± 0.3	2.1 ± 0.3	5.5	0.211
*Nucleus accumbens*	0.8 ± 0.2	1.0 ± 0.1	13.9	**0.007**

Results of the multivariate analysis of deep grey matter structures volumes across the study groups with BMI as covariate. Volumes are reported divided by normalized brain volume (NBV)

*STD* standard deviation

Values of *p* < 0.05 are considered statistically significant and are marked in bold

Linear regression analysis indicated significant associations of duration of boxing in years and right thalamus volume (β = −0.811, *P* = 0.049). Effects regarding right globus pallidus almost achieved significance with (β = −0.765, *P* = 0.066). The predictor variables exhibited no significant effects on the remaining volumetric measurements in boxers, see Table 4. Also, there were no significant associations between number of fights and volumetric measurements.

**Table 4:** Linear regression analysis of deep grey matter structure volumes and years of boxing

Volumetric structure	Years of boxing β‑value	*P*-value
*Right thalamus*	0.811	**0.049**
*Left thalamus*	−0.0623	0.165
*Right caudate nucleus*	0.050	0.904
*Left caudate nucleus*	0.222	0.545
*Right putamen*	−0.062	0.897
*Left putamen*	0.016	0.972
*Right globus pallidus*	−0.765	0.066
*Left globus pallidus*	−0.385	0.40
*Right amygdala*	−0.617	0.131
*Left amygdala*	−0.019	0.966
*Right nucleus accumbens*	0.058	0.897
*Left nucleus accumbens*	0.113	0.815

Effect of years of boxing on deep grey matter structures

Values of *p* < 0.05 are considered statistically significant and are marked in bold

## Discussion

We investigated differences in deep grey matter structures between amateur boxers and healthy control subjects. A significantly lower volume for amateur boxers was found in the thalamus, the caudate nucleus, the putamen and globus pallidus as well as the nucleus accumbens, but not the amygdala. The largest significant volume difference of 13.9% between amateur boxers and healthy controls in absolute numbers, was detected in the nucleus accumbens, closely followed by the caudate nucleus with a difference of 11.1% and the globus pallidus with a difference of 9.6%. Bendlin et al. showed a decline in volume over the course of a year after the acute mTBI event affecting, among others, the nucleus accumbens, the putamen and the thalamus [33].The volume loss of basal ganglia and the nucleus accumbens was in line with previous studies in moderate to severe TBI [33–38] and has been associated with a progression of neurodegenerative diseases [39, 40], also comprising Parkinson’s disease [41, 42]. Thalamic volume reduction was also apparent in our boxer study group, which supplements the finding of previous studies in TBI [34, 35, 43]. It is noteworthy that head collusions were replicated in a biomechanical investigation using finite element modelling and revealed that the thalamus and midbrain experience the highest shearing stress [44]. The thalamus serves as a sensory relay center which transfers information from the limbic system, basal ganglia and cerebellum to the cerebral cortex [45]. We can assume that this atrophy is attributed to the large number of white matter tracts connected to the thalamus. In the present study, we did not find a significant volume difference in the amygdala volume between our study groups, this is opposed to previous investigations in TBI [36, 46]. On the other hand, the lack of significance might be due to a small study sample in our study.

Of note is our finding that TIV was lower in boxers than in healthy controls. Yet, we are hesitant to draw conclusions of the possible causes. Many potentially confounding factors, among them differences in upbringing, nurture and genetic profile between boxers and healthy controls are imaginable and might influence head size and total intracranial volume.

In mTBI, cortical GM atrophy is more likely to be happening secondary to underlying WM damage, possibly due to retrograde cell death after axonal injury rather than to damage acquired from the direct impact on brain parenchyma. This might explain the time delay in the detection of cortical GM loss before the occurrence of microstructural changes in WM. Several publications showed in longitudinal studies that degenerative adaptation of the brain takes place long after the acute injury, resulting in volume loss of WM and then GM, preceded by an increasing derangement of WM microstructural integrity measurements [33, 47, 48]. Markers for disturbances of WM microstructural integrity are decreased fractional anisotropy (FA) and increased mean diffusivity (MD) in WM structures and were found to be associated with traumatic brain injury in mTBI [49, 50]. It should be noted that the amateur boxers in our study would have been excluded if their brain scans revealed any signs of visible cortical or subcortical contusions, so we assume that the differences in deep GM structure volumes reflect chronic brain damage. Also, in a previous publication, examining a cohort from which our boxers and healthy controls were derived, only 3 of 42 boxers included in that study showed microbleeds that might be associated with traumatic brain injury [17]. Therefore, only a small fraction of the boxers in the study showed traumatic brain changes visible in our conventional MRI protocols.

We found that years of boxing had a significant association with the reduction of right-sided thalamic volume in boxers. This is in line with previous prospective studies that reported greater decline in thalamic volume with increased fight exposure [15, 51]; however, unlike in the aforementioned study by Bray et al. we were not able to test the interaction between weight class and fight exposure regarding different brain volumes due to our small study group. We were also not able to reproduce other significant effects on the remaining brain volumetric measurements, such as the right putamen as reported by Bray et al. In our analysis the number of fights did not predict volumetric differences.

In the study cohort of Bendlin et al. patients after traumatic brain injury even improved in their neuropsychological test performance between 2 months and 1 year post-injury [33], suggesting an acute neuropsychological deterioration after the injury, which can be compensated for after some time despite the progression of microstructural and macrostructural changes. This is in line with the lack of significant differences in neuropsychological measures between our amateur boxer and healthy control group. As we did not consider the timing of potential traumatic brain injuries in the scheduling of our MR imaging and neuropsychological testing appointments, we assume that most boxers in our study group were not most recently affected by traumatic brain injury but rather exposed to potential mTBI over the time of boxing years which might be a mostly compensated state. Nevertheless, other longitudinal studies correlated brain atrophy in mTBI [52] to poorer vocational outcome, as well as in moderate to severe TBI to increased disability and worse neuropsychological outcome [46]. It is an intriguing paradox that even though the brains of the amateur boxers showed seemingly disadvantageous differences, namely deep GM volume loss, neuropsychological testing could not distinguish the boxers and controls in our study. This might be due to methodological issues, such as the heterogeneity of neuropsychological tests or there must be some brain mechanisms that compensates, at least in the beginning, the worsening of neuropsychological functioning.

### Limitations

While our study provides some important insights into the brain of amateur boxers, exposed to mTBI, there are several limitations. First, as discussed above, time of injury has an impact on volumetric as well as neuropsychological results, but the time of potential injury was not assessed in our amateur boxer group which might add inaccuracies in the data. Instead, we assumed continuous possible injuries acquired over time of boxing years and only excluded the occurrence of moderate and severe traumatic brain injury via the assessment of conventional MRI imaging and questioning the boxers about the occurrence of long-lasting neurological deficits after fights in the past. Another important caveat is the lack of longitudinal imaging and neuropsychological data acquisition, which limits our ability to understand causative association between mTBI and regional brain atrophy as well as the timing of the atrophy progress. Furthermore, the study could have benefitted from a larger sample size, which especially for the regression analysis might have led to higher statistical power and revealed more associations between volumetric changes and behavioral measures.

## Conclusion

In this study exploring the brains of amateur boxers, our findings indicate that atrophy is not a uniformly diffuse process that affects WM and GM, but rather has predilection for various subcortical structures which may be more vulnerable than others, such as nucleus accumbens, thalamus, caudate nucleus, putamen and globus pallidus. Further studies will be needed to determine the impact and progression of regional atrophy on particular neuropsychological outcomes, and it would be worthwhile to incorporate the volumetric data with parameters of axonal integrity in order to understand the relationship between acute white matter injury and volume loss in adjacent grey matter.
